# American Association of Obstetricians and Gynecologists

**Published:** 1897-10

**Authors:** 


*Proceedings of the Tenth Annual Meeting, held at Niagara Falls, N. Y., August* 17–20, 1897.

### First Day—Morning Session.

THE Association met at the Cataract House under the presidency of Dr. James F. W. Ross, of Toronto.

An address of welcome was delivered by Dr. W. R. Campbell, of Niagara Falls, which was responded to by the President.

The first paper was read by the Secretary of the Association, Dr. William Warren Potter, of Buffalo, entitled

#### PRINCIPLES OF TREATMENT IN PUERPERAL ECLAMPSIA.

The author, at the outset, referred to the views advanced by M. Charpentier at the Geneva Congress last year, relative to the treatment of puerperal eclampsia, especially as to the applicability of the induction of premature labor for its relief and which he said was so at variance with those that he had been accustomed to entertain and the verity of which he had so frequently tested, that he confessed surprise thereat. It was not in any spirit of controversy that he ventured at this time to oppose M. Charpentier's teachings, but simply in the interest of professional progress and science. The author then gave his own views regarding this subject, saying that they were fortified by an experience and observation extending over a period of many years. Furthermore, in the application of the principles he would lay down and advocate, a measure of success had been obtained at his hands that did not present itself under a contrary method previously pursued. The chief object of the paper was to advocate principles which the speaker grouped under the following heads:

1. Though the pathogenesis of eclampsia is unsettled it belongs solely to the pregnant or puerperal state. It is not apoplectic, epileptic or hysteric in character.

2. It depends upon toxemia due to overproduction of toxins and under-elimination by the emunctories.

3. These toxins probably have their origin in the ingesta, in intestinal putrefaction, in fetal metabolism, one or all, and there is coëxisting sluggishness, impairment or suspension of elimination.

4. When the prodromes of eclampsia appear, the kidney should be interrogated as to its functions and all symptoms carefully watched.

5. Treatment is preventive and curative. Preventive treatment is medicinal and hygienic; curative treatment is medicinal and obstetric.

6. Milk diet and distilled water should be given in the pre-eclamptic state to dilute the poison, hasten its elimination and nourish the patient.

7. Bloodletting should only be employed in plethora or cyanosis. It is liable to cause anemia if persisted in or repeated, whereas red blood corpuscles must be conserved, not wasted. Glonoin diminishes vasomotor spasm, hence may be given freely in appropriate cases. Veratrum viride is a cardiac depressant and a dangerous remedy if pushed to an extent that will control convulsions.

8. Eclampsia is the expression of a further maternal intolerance of the fetus; hence, as a prime measure, the uterus should be speedily emptied of its contents.

9. Medicinal treatment alone is delusive and, when relied on exclusively, is fraught with danger to both mother and fetus; whereas in the prompt induction of labor is found a rational application of science to a desperate condition.

10. Finally, it furnishes, in the present state of our knowledge, the only basis of expectation for a diminished mortality in a toxemic disease of high death-rate.

Dr. John M. Duff, of Pittsburg, opened the discussion and said, relative to the induction of premature labor in cases of eclampsia in which albuminuria is present, that conscientious practitioners would not hesitate, although in exceptional cases careful attention to medicinal treatment would cause the albumin to disappear and the patient would go on to normal labor. As a diuretic, he had secured excellent results from the use of milk, with a spoon, as hot as it could be borne by the patient.

Dr. H. W. Longyear, of Detroit, was surprised that the essayist did not mention the necessity of inducing premature labor before the occurrence of eclamptic symptoms. Such treatment appeared analogous to snuffing out the light of a fuse after an explosion had occurred. Premature labor should be brought about in every instance in which a daily examination of the urine showed a constantly increasing amount of albumin, with prodromal symptoms of toxemia. During the past year he had induced premature labor twice under these circumstances. In one case the patient was seven and a half months pregnant and in the other eight months. He would not advocate this course merely because albumin was present in the urine, but because of this and the fact that the amount of urea eliminated was constantly decreasing. Hot milk was very valuable, in his opinion, in the treatment of these cases.

Dr. Walter B. Chase, of Brooklyn, stated that the decrease in the daily amount of urea was a most valuable indication of the probable occurrence of toxemia in these cases. Unfortunately, many eclamptic patients were seen for the first time only when in convulsions and then the induction of premature labor was the single measure that could be relied on. In certain cases, however, in which the urine clears after a few days, it was unjustifiable to end the period of gestation prematurely. The best method of stopping the convulsions, particularly when arterial pressure is high, is by the administration of veratrum viride.

Dr. W. H. Wenning, of Cincinnati, doubted the advisability of bringing on premature labor under all circumstances in which toxemic symptoms develop. In many cases, if the os was rigid and not dilated, there was danger of inducing convulsions in the attempt of the obstetrician to dilate. He considers the psychologic element an important one. He gave the history of a patient who presented no symptoms whatever of toxemic infection, there being no albuminuria or edema present, but who died shortly after the birth of her child. Another patient fearing that her child might be born before the expiration of nine months from the date of her marriage, was in a state of constant nervousness on this account and when labor finally took place forceps had to be used. The patient soon developed a condition of marked apathy and would pay no attention to her child. On six different occasions she had a convulsive seizure immediately after the physician's visit and just as he was leaving the house, but each immediately ceased on his being recalled. The patient finally died, although there had been no true symptoms of toxemia.

Dr. Charles Stover, of Amsterdam, N. Y., said an important point was, how soon can the condition of puerperal eclampsia be diagnosticated ? He emphasised the importance of a daily analysis of the urine. If the physician is called to see a patient late in the progress of the case, the urinalysis might present entirely different features from that in the beginning. He knew of three cases which occurred during the past three months in which the attending physician had waited for more marked symptoms to occur after albuminuria had developed and each of the patients died. He referred to the method of Dr. James H. Etheridge, of Chicago, of estimating the amount of urea and considered it a valuable one. He also spoke of a case which came under his own observation, in which but 500 grains of urinary solids were excreted daily when there should have been 1,300, the patient developing eclampsia.

The President, Dr. James F. W. Ross, said that he had found that the occurrence in females during early life, of exanthematous fevers, had a marked influence on subsequent renal insufficiency ; and in more than one instance he had been able to trace this insufficiency to an attack of scarlet fever or diphtheria occurring during childbirth. The question of puerperal eclampsia was still unsettled; its pathology was shrouded in darkness and a step forward would be for the larger medical associations to institute a series of investigations with the hope of determining its cause. In many of the worst cases premonitory symptoms were not present at all. He considered it unwise to use haste in emptying the uterus, when this has been determined on, as there was great danger of lacerating the parts. The question as to the advisibility of inducing premature labor in the case of a patient who in a former gestation had eclamptic attacks, but in whom at the time of the subsequent pregnancy no symptoms of toxemia were present, was an important one. The speaker had terminated the period of gestation in a number of cases of this sort and believed such action entirely justifiable, although it cannot be denied that a woman might go through a number of pregnancies normally before the occurrence of a second series of eclamptic seizures.

Dr. Longyear—Would you advise the induction of premature labor under these circumstances if there are no symptoms whatever of renal insufficiency ?

President Ross—Yes. (The President then reported a case in which he had practised it.)

Dr. Rufus B. Hall, of Cincinnati, looked upon such a doctrine as extremely dangerous and strongly opposed it. Simply because a woman at some previous time had had eclampsia was not sufficient reason for terminating gestation prematurely when no symptoms of toxemia were present.

Dr. POTTER, in closing the discussion, said the occurrence of albuminuria was a danger signal, but that decrease in the daily amount of urea excreted by a pregnant woman was still more valuable as a prodromal sign. The violent production of premature labor was never justifiable. If eclamptic attacks occurred some time previous to labor the physician might take time in emptying the uterus, but in the intrapartum variety, the cervix should be carefully dilated by means of a steel dilator and the hand and, if necessary, forceps applied and the fetus delivered. He had accomplished this within three hours when the cervix was rigid, without lacerating the tissues and had saved the life of both mother and child.

Dr. H. W. LONGYEAR, of Detroit, read a paper on

PUERPERAL DIPHTHERIA, the object of which was to bring before the profession the fact that the Klebs-Löffler bacillus is a potent factor in the etiology of puerperal infection, six cases being reported in proof of the position of the writer. The diphtheritic character of each case was determined not only by clinical evidence, but by bacteriologic examination made by the bacteriologist of the Detroit Board of Health. Five of the cases recovered and one died, the one death being the only case in which antidiphtheritic serum was not used.

The writer recommends bacteriologic examination in all cases of puerperal infection, condemns curetting in all cases where a membranous exudate is present, and recommends the use of antidiphtheritic serum, the intrauterine application of a strong solution of iodine and carbolic acid at the beginning of treatment, drainage of the uterus by a tube, intrauterine irrigation with antiseptic solutions, bi-hourly vaginal injections of hydrogen peroxide, and the usual supportive treatment with quinine and whisky. In mixed cases of Klebs-Löffler bacillus and streptococcus he would first use the antidiphtheritic serum, and then in twenty-four to forty-eight hours begin the use of the antistreptococcic serum. He believes that the true character of this form of infection has not been heretofore recognised, but that it will be found to be more or less prevalent in all localities in which diphtheria exists if sufficiently critical examinations are made. The physician and midwife are warned to use especial care in such localities, as the post-partum parturient canal furnishes the most favorable soil for the growth of the Klebs-Löffler bacillus.

Dr. JOHN M. DUFF, of Pittsburg, followed with a paper on

THE SOURCE OF PUERPERAL SEPSIS.

The source of puerperal sepsis, he said, was given by the authorities as contagion from a woman similarly affected, from suppurating or decaying tissues, from putrefying substances within or without the body; and from zymotic diseases, especially erysipelas and diphtheria.

The exact source in any given case was not always easy to determine. In a large proportion of cases, however, a careful inquiry would be rewarded with the revelation of a source. When found it generally proved to be one which could have been avoided.

Aseptic midwifery had done much to prevent puerperal sepsis, and consequently to lower the rate of mortality following childbirth. He had heard and read remarks by members of the profession in sentiment expressing the opinion that the accoucheur who was so unfortunate as to have a case of puerperal sepsis occur in his obstetric work was guilty of malpractice. Such expressions he thought were extravagant and were not warranted by our present knowledge and experience. Sepsis does occur sometimes despite the best efforts of the practitioner to prevent it. While he believes puerperal sepsis cannot always be prevented, he does believe that with ideal surroundings, with a careful and skilful physician, supplemented by an educated and conscientious nurse, the number of cases could be reduced to a minimum. His observations teach him that while on the one hand there was no attempt at aseptic or antiseptic precautions, there was frequently too much reliance placed upon antiseptics. For instance, a well-known member of the profession had said in his hearing a short time since that he felt perfectly safe in going to attend a case of labor after waiting upon a case of erysipelas or diphtheria, if he washed his hands well in a strong bichloride solution. Dr. Duff thinks his patients will be safer if he went to them under such circumstances with fear and trembling, which would cause him to perform further ablutions if possible.

Without discussing at length the different known sources of infection or defining the varieties of sepsis, the author reported fifty cases of puerperal sepsis that had come under his observation.

These two papers were then discussed together.

Dr. A. Goldspohn, of Chicago, said such papers as the one presented by Dr. Duff were calculated to do a great deal of good. The old idea was still prevalent that puerperal fever was something specific. This was a mistake. It was an infection, just the same as a phlegmon or other inflammation which occurs in any other part of the body. The cause of it was thoroughly settled, viz., septic germs. Men who had labored exhaustively in Europe on this subject had come to the conclusion that the most dangerous germ of the disease was the streptococcus, which was present in about 4 per cent. of the cases.

Dr. Lewis S. McMurtry, of Louisville, said physicians were apt to think that because puerperal sepsis had been discussed a number of times and so much had been written about it, that professional sentiment was crystallised and that it was a work of supererogation to agitate the subject, but if we would look around us in our respective localities we would find that the number of deaths from puerperal sepsis is very large. He sometimes thought that we do not realise at the present time, in large cities and in country districts, how great the mortality is from this altogether preventable disease, and until the mortality is reduced very materially the subject should be discussed repeatedly and published to the profession, showing the responsibility and duty that rest upon practitioners.

Dr. Charles G. Cumston, of Boston, spoke of the principal cause of puerperal sepsis from a pathologic standpoint. In 1893 he conducted a series of experiments with reference to the bacterium coli. Among the specimens and autopsies that he performed in view of ascertaining the virulence of this organism he had five cases of puerperal septicemia, in all of which the uterus was carefully examined. The folds of the membrane lining the uterine cavity contained the bacterium coli, as was subsequently proven by cultures. Two of the uteri were removed by the vaginal route and on the other three patients he performed autopsies. He could not give the clinical history of the cases, but only the anatomic and bacteriologic findings. A second cause of puerperal septicemia and a potent one was the gonococcus, in that it prepares the way for pus-producing organism to enter the uterine cavity and thus set up infection.

Dr. James F. Baldwin, of Columbus, Ohio, stated that in three cases of so-called puerperal fever that had come under his observation the infection was found to be due to appendicitis and he supposes it was attributable to the bacillus coli communis. Two of the cases were operated on and recovered. The third case was operated on by another surgeon and died.

Dr. Wm. Warren Potter, of Buffalo, drew attention to the point that in discussions on this question as presented to societies and even in magazine articles, there was a disposition to drop from the literature of the subject the term puerperal fever. There were some old fashioned things that the profession need to cling to because they were good, but puerperal fever was no longer recognised as a distinct entity. The term was misleading in itself, because it teaches an erroneous pathology and points to an equally erroneous treatment.

Dr. Adam H. Wright, of Toronto, said that in the majority of cases of puerperal sepsis the poison comes from without and the condition can be avoided. It is a preventable disease.

Dr. Edwin Walker, of Evansville, Ind., said that it was a much more rational thing to dry out the genital tract than to wash it out. He would admit that even if the genital tract was dried out we could not get rid of all the germs, but we could deprive them of their nourishment. If we use a douche of clean water we furnish the germs a pabulum for their nourishment. In puerperal sepsis he would advise wiping out the genital tract rather than douching it.

President Ross said he had seen a large number of cases of puerperal sepsis in consultation, owing to the fact that the patients had either pus tubes, pelvic abscess, or some such condition present, although he did not do an obstetric practice. He gives the causes of puerperal sepsis as follows: (1) gonorrhea; (2) retained placenta or membranes; (3) dirty hands and instruments; (4) lacerations of cervix and perineum, improperly repaired or protected; (5) intra-abdominal disease or tumors.

Dr. Albert Vander Veer, of Albany, said he was quite sure that he reiterated the experience of other abdominal surgeons when he said that it was sad to witness the amount of ignorance that existed in regard to careful aseptic obstetric work. If we are to use serum therapy, it is very essential to make a differential diagnosis, as was touched upon by Dr. Longyear, and the speaker fancied that the future history of this work would be in the direction of the serum of streptococci. The cases must be seen as early as possible.

Dr. Albert Goldspohn, of Chicago, said the gonococcus was not the exciting, but merely the predisposing cause of puerperal sepsis. There was no case of puerperal sepsis on record in which the gonococcus alone was the cause of the trouble. Other pus microbes were present in this condition and the infection was a mixed one.

### First Day—Afternoon Session.

Dr. Adam H. Wright, of Toronto, read a paper on

#### TOXEMIA OF PREGNANCY, in which he said that the chief symptoms of this condition were salivation; disorders of digestion, with sometimes a peculiar taste and constipation; general malaise; anemia; nervous disturbances or headache; disorders of vision; irritability; deficient excretion of urine or some of its constituents and albuminuria. To speak briefly, he thought any sign of the slightest departure from ordinary health during pregnancy should make us suspect the advent of general toxemia and should receive careful investigation and thorough treatment. If, for instance, there be general malaise, with slight headache, but no albumin in the urine, let us not be deceived, since albuminuria was one of the symptoms of systemic poisoning and sometimes the last to appear. Its absence proved absolutely nothing.

Coming to the treatment, reference was made to milk diet for toxemia or albuminuria of pregnancy. He believes that a purely milk diet is good for young babes and calves, but he does not think that it is suitable for adult human beings. Yeo, in his admirable book on Food in Health and Disease, shows clearly that milk is not a suitable food for healthy adults, because it contains an excess of albuminates and fats and that it should be mixed with other foods, especially the carbohydrates. If it be conceded that milk alone is not the best food for healthy adults, it is difficult to conceive how it can be the most suitable in any case of disease.

In connection with his results in the Burnside Hospital, in nine years they have had sixty-five cases of toxemia with albuminuria with two deaths, both from eclampsia. One of these two patients came into the hospital in a dying condition with eclampsia, having received no previous treatment. In the sixty-five cases mentioned there were many cases where the albuminuria and other symptoms were only slight.

In conclusion, the author enumerated the main points in his treatment as follows: (1) a carefully selected mixed diet with plenty of water, plain and mineral, lemonade without sugar and the like; (2) rest, good hygienic surroundings and proper clothing; (3) the regular and persistent use of purgatives for weeks or months, with a preference for epsom salts; (4) a warm daily bath; (5) the induction of abortion or premature labor in rare cases.

### THE TREATMENT OF PUERPERAL ENDOMETRITIS BY THE CAROSSA METHOD.

By Edward J. Ill, M. D., Newark, N. J.

There appeared a pamphlet early in the winter of 1896, by K. Carossa, describing a method which consists simply in the use of alcohol as an irrigating material, supplemented by gauze packing of the uterus in such a way that the alcohol finds its way into the most distant recesses of the uterus. A catheter is introduced into the uterus and this organ filled with absorbent gauze in a lightly but thorough fashion. At the external end of this catheter a funnel is attached, through which a 20 to 25 volume per cent. of alcohol solution is poured so as to flow into the gauze with which the uterus is filled. The quantity to be used is from 30 to 50 c.c. every hour, day and night. In three to six days the gauze is removed.

The originator of this method presents some fantastic theories of the evaporation of alcohol which the author of the paper cannot agree with and to which Dr. Carossa attributes his results. Dr. Ill has used the above method, slightly modified, with good results and recommends it for further trial, especially so on account of its great simplicity.

Dr. Rufus B. Hall, of Cincinnati, read a paper entitled

### SEQUELÆ FOLLOWING SUPRAVAGINAL HYSTERECTOMY.

He said he thought the profession has been too hasty in approving the present methods. It had taken the stand that there was nothing more to be desired; the operation was complete. While the author had no new method to offer, he hoped the discussion of his paper would suggest something to improve the technique of the present methods.

He referred to objections to the extraperitoneal method and then spoke more in detail of total extirpation. He was one of the first to advocate and make this operation and he had attained excellent results with it. However, it did not meet his ideas of a perfect operation. The final results were good, but there was a primary difficulty that was disagreeable. Suppuration necessarily took place about the ligatures on the sixth or seventh day, causing a slight rise in temperature. To overcome the suppuration, he used specially prepared catgut for the ligatures below the peritoneum. He found the catgut unreliable on account of the danger from hemorrhage through slipping of the knots and so abandoned its use.

After seeing Dr. Kelly, of Baltimore, in 1895, make the supravaginal operation with his modifications in the technique of the Baer method, Dr. Hall adopted that method. He operated according to it forty-six times, but was not pleased with his results. Eleven of the cases had post-operative sequelæ, due to the buried ligatures. All the patients made good primary recoveries, only one showing any signs of trouble earlier than the seventh week. This one had a discharge of pus from the vagina at the end of the fourth week. The discharge disappeared to return again at intervals of a few days to four or five weeks, and only ceased when the ligatures were removed. The other cases had similar histories, except that they did not have any trouble until from four to eleven months after the operation. Within a few days after the ligatures were removed, the patients were well and have remained so, except two. These two, one sixteen and the other eighteen months since the operation, are still having trouble as the ligatures have not all come away. The author said he knew the material was not at fault or there would have been trouble immediately following the operation. Besides, the same material was used in other operations and no trouble resulted. He said he believed a large percentage of the patients so operated on would certainly have this trouble. The general surgeons are abandoning the use of silk, silkworm gut and silver wire in Bassini's operation for this very reason, and he felt it only logical for the gynecologist to expect to have to do the same. He urged the other gynecologists to tell their results with this method and compare notes.

The author closed his paper by saying that in spite of the fault he had to find with total extirpation, he preferred it to supravaginal hysterectomy as practised by Baer and modified by others. He felt more certain of his final results with the former method.

Dr. George M. Hughes, of Philadelphia, read a paper on

THE SEQUELÆ OF DEAD LIGATURES AND SUTURES.

While assisting Dr. Joseph Price in his abdominal work the essayist had recently been greatly interested in a number of cases in which he reopened the abdomen for the freeing of adhesions and the removal of dead ligatures and sutures. Sutures and ligatures should be of that material which is easiest sterilised and which combines great strength in a small bulk. It was always preferable to use a material capable of being rendered aseptic by heat or boiling. If this could be done, we could at all times have the means at hand to render our ligatures perfectly sterile. For this purpose he finds that for pedicle ligatures and for bowel work the twisted Chinese silk is the best, of finest quality and sufficiently small to secure safe tying; for closure of the abdominal incision silkworm gut and the through-and-through method. What becomes of the ligatures? If small and sterile, they become encapsulated and rapidly absorbed. If plaited ligatures and large hawsers are used, whether infected or not, they are never absorbed, but their presence as foreign bodies give a train of symptoms unbearable in their distress and constant in their duration. The same conditions are found about the pedicles, only here we have the adhesions of omentum, large and small bowel and bladder to both the pedicles. To obviate post-operative sequelæ we should select that method of applying ligatures which gives safety with the least quantity of material. For pedicles the simple figure-of-eight tie is the best, in that it gives a firm, small, strong tie and one not liable to slip. The pedicle is then cut cone-shape. The pedicle must be made as small as possible; large pedicles are prone to behave badly, and to this is due the post-operative adhesions of omentum, bowel or bladder. To illustrate his remarks the essayist selected and reported five instructive cases.

### Second Day—Morning Session.

### POST-CLIMACTERIC CONDITIONS THAT SIMULATE ADVANCED UTERINE CANCER.

A paper on this subject was read by Dr. M. Rosenwasser, of Cleveland, Ohio. The writer commends the teaching that irregular hemorrhages and sero-sanguineous discharges, whether occurring during the parturient stage or long after the menopause, are good and sufficient reasons to suspect malignancy. We carefully watch for early symptoms and by their detection occasionally succeed in removing the disease while it is still local. On the other hand, we sometimes are caught off our guard when confronted by post-climacteric cases presenting all the classic characteristics of advanced malignant disease. Without the same circumspection exercised in the early stages we thoughtlessly pronounce the case beyond remedy or hope, specifying even the extreme possibility of life. The text-books are deficient in not sounding a note of warning against possible errors in the late stages. The so-called classic symptoms may be due to other (not malignant) conditions of the genital tract. Owing to effacement of the vaginal portion of the cervix in old age the differential diagnosis is in most cases limited to corporeal diseases of the uterus. Before the diagnosis of corporeal cancer can be made, other diseases must be excluded.

The conditions which are likely to simulate advanced cancer are the following: (1) senile vaginitis; (2) foreign bodies in the vagina; (3) gangrenous fibroids; (4) atrophic, senile or post-climacteric endometritis; (5) post-climacteric pyometra.

Of these conditions the last is especially liable to lead to errors. The writer gave the details of a case of pyometra occurring recently in his own experience and submitted abstracts of five more or less similar instances found scattered in the literature of the past seven years. In all these cases either a positive or provisional diagnosis of corporeal cancer had been made.

In conclusion, the author called attention to the singular fact that in the presence of the essential conditions—age, low vitality, cicatricial tissue, adhesions, chronic inflammation and irritating discharges—cases of transformation into malignant disease are either unknown or exceedingly rare.

Dr. Richard Douglas, of Nashville, Tenn., followed with a contribution on

### CERTAIN CYSTS OF THE ABDOMINAL WALL, in which he confined himself to a consideration of abnormalities of the urachus, and reported an interesting case of urachal cyst which occurred in a woman 36 years of age, married eleven years and sterile.

### CONSERVATION OF THE OVARY.

A paper on this subject was read by Dr. B. Sherwood-Dunn, of Los Angeles, Cal. The author said that Brown-Séquard believed and taught as a principle of physiology that every gland, whether or not provided with excretive ducts, gives to the blood a certain useful principle, the absence of which is felt and made apparent after their extirpation, or the destruction or modification of their functional activity by disease. The recent publication of researches made by Mond and Chrobak, of Vienna, Jayle and Lissac, of Paris,

Mainzer, of Berlin, and Muret, of Lauzanne, had given definite form to certain ideas that the speaker had conceived upon this subject, born of a series of observations taken in his hospital service in Paris of a variety of troubles and functional disturbances which more or less constantly follow as a result of double oöphorectomy. These various troubles and functional derangements, which are constant, although variable in degree, in women who have had the menopause anticipated by castration, form to his mind one of the strongest arguments in support of the glandular theory.

From observations made upon 100 cases operated upon in Broca and St. Louis Hospitals, in Paris, he found that where the woman had prematurely lost both ovaries 78 per cent. subsequently suffered a notable loss of memory; 60 per cent. were troubled with flashes of heat and vertigo; 50 per cent. confessed to a change in their character, having become more irritable, less patient, and some of them so changed as to give way to violent and irresponsible fits of temper; 42 per cent. suffered more or less from mental depression and 10 per cent. were so depressed as to verge upon melancholia. In 75 per cent. there was a diminution in sexual desire and some of these claimed they experienced no sexual pleasure; 13 per cent. were not relieved of the pain from which they suffered; 35 per cent. increased in weight and some became abnormally fat. Some complained of a diminution in the power of vision; 12 per cent. noted a change in the tone of their voice to a heavier, more masculine quality. Some 15 per cent. suffered from irregular attacks of minor skin affection; 25 per cent. had severe headaches, as a rule increased in intensity at the menstrual period. Equally as many complained of nightmare, more or less constant, while about 5 per cent. suffered from insomnia. In a few cases there existed a sexual hyperexcitability not present prior to the castration. He particularly noted a few cases presenting gastric reflexes, where without any premonitory symptoms or apparent cause the stomach would reject food or refuse to prepare it for intestinal digestion and the consequent distress following the fermentation compelled the patient to seek relief. It should be noted that usually these troubles were more marked in women under 30 or 33 years of age.

He had been favorably disposed to the hypothesis advanced by Brown-Séquard for some time, and any scepticism that he may have entertained of the theory of ovarian secretion and its usefulness and necessity to the equipoise of the whole system has been completely dissipated by the results of experiments made with ovarian substance, or ovarin, in patients who have lost both ovaries or were suffering from troubles which, in a greater or less measure, were due to a diseased condition of the ovary. In the observations of the authorities mentioned use was made of: (1) the ovary in its natural state; (2) the desiccated and powdered organ; (3) glycerin extract of the ovary; (4) liquid ovarin. The first form presents two objections, viz., difficulty of obtaining the fresh organ and greater difficulty in getting the patient to take it. The second form of powder has been the one most favored by all and has given as good results as the administration of the hashed fresh ovary or of the glycerin extract or the hypodermic injection of liquid ovarin, prepared after the method of Brown-Séquard and preserved in sealed tubes. The essayist has so far used only the powder and the tablets, the latter in only one case, which did not respond as quickly as the others and he soon changed to the powder. But he would not say that one is better than the other, as he had not given the tablets a fair trial, neither could he expect such good fortune as that the good effects observed in his three cases will continue in those that follow. The author closed with the report of three interesting cases.

Dr. James F. W. Ross, of Toronto, delivered the President's address. He selected for his subject

SURGERY AND FACTS.

He said the work of the association was confined between the diaphragm, the perineum and the abdominal walls and that the members had met to cultivate and promote a knowledge of whatever relates to abdominal surgery, obstetrics and gynecology. Attention was drawn to some unsettled questions, the first being peritonitis. Are we able to do more to save the lives of patients suffering from this disease in its acute form than we were ten years ago? Are we not but little better off with all our antiseptic and aseptic washes, gauze and tube-drains and purgatives? He was satisfied that surgery could carry us no further when battling with this disease. The question of operating upon the appendix and the diagnosis and treatment of ectopic gestation had been fairly well settled. The method of dealing with the pedicle in ovariotomy had been settled, except for the fact that some operators preferred silk, while others were assured of the safety of catgut. Operations upon the gall-bladder and gall-ducts had been performed many times during the past ten years and they were now well recognised as proper surgical procedures. The operations of nephrectomy and nephrotomy were looked upon as everyday procedures, justified by the consensus of surgical opinion. Abdominal hysterectomy was an operation that had been much improved and simplified, some operators being still wedded to the clamp, while others preferred some of the other methods. The advisability of oöphorectomy for some fibroids could not be doubted. There were two operations performed that Dr. Ross thought are of doubtful value, viz., the fastening of the kidney to the side and the fastening of the uterus anywhere. He considers that these operations have been recklessly performed and unnecessarily done. Another class of operations to which more thought must be given were those for intraäbdominal and intrapelvic cancer. When peritoneal cancer has been diagnosticated, surely exploratory operation is uncalled for. Operations for other forms of cancer are of questionable utility. Take, for instance, resection of the cancerous intestine, gastroenterostomy for pyloric cancer and removal of the uterus for uterine cancer. Gastroenterostomy and resection of the intestine are poor makeshifts. For carcinoma uteri, vaginal hysterectomy is the only operation that should be contemplated, but at best it prolongs life but a short time.

### Evening Session.

### SOME OBSERVATIONS UPON VENTRAL FIXATION.

By Dr. Herman E. Hayd, of Buffalo.

In this paper it was stated that ventral fixation or suspension of the uterus, coupled with the various plastic operations upon the cervix and vagina is the only means, surgically or anatomically, which will fix and support for future comfort and well-being an extremely prolapsed uterus. However, because a uterus sometimes offers a serious impediment to delivery by interfering with the proper dilatation of the organ is no reason why the operation should be relegated to oblivion. *Per contra*, it should be employed to relieve that large class of suffering women who have passed beyond the child-bearing period and who most frequently are the victims of extreme procidentia uteri. In his last six cases of ventral fixation he sewed the uterus to the abdominal wall with chromocised catgut (No. 3) and did not even scarify the peritoneal covering of the uterus. He held the organ by thin sutures, which took in simply the peritoneum and the connective tissue over it; but in one case where the organ was very heavy and the woman short and stout he hitched on to the rectal fascia and muscles. He has invariably sewed the anterior surface of the uterus, feeling more satisfied with the position assumed under these circumstances than where the sutures catch the superior and posterior surface, as is advocated in ventral suspension. In the cases he has operated on and those he has examined after operation of ventral suspension he has found the organ in a too anteflected position and it may be the cause of some future annoyance. He has discarded silk and all unabsorbable ligature materials, believing that catgut can be sterilised and rendered absolutely safe and is perfectly manageable.

Dr. C. C. Frederick, of Buffalo, N. Y., read a paper entitled

WHICH IS THE PREFERABLE OPERATIVE METHOD OF HOLDING THE UTERUS IN POSITION?

All retroversions do not produce symptoms, but a certain proportion are accompanied by hypertrophy of the uterus, endometritis, leucorrhea, pain, backache, menorrhagia, metrorrhagia or general malaise. Constitutional treatment fails to relieve a large proportion of them without restoration of the uterus to a normal position and a cure of the accompanying hypertrophy and endometritis by reposition. The large factors in the continuance of the ill-effect of retroversion are torsion of vessels, infection of the endometrium and defective drainage of the uterine cavity. Retroversion is a first stage of prolapse and ought, for that reason alone, to be replaced and held in position. Sterility is one frequent result of retroversion. Results of treatment are good by operation or by holding the uterus in position by a pessary, if possible, together with general tonic treatment, Weir Mitchell rest treatment and the like. A large proportion of cases are cured and eventually get strong and well again. Ventrofixation has been discontinued by the writer in women liable to bear children. He uses it only to hold up the uterus in operation for prolapse and in those cases where he has removed both tubes and desires to hold the uterus in position, time being an element in the operation. The writer has seen no ill results during labor and knows of none occurring in his own cases, although several have borne children. He has seen no recurrence of retroversion even in those who have borne children. His preference is given to the Alexander operation or some of its modifications in cases of women who have borne children and there are no adhesions or diseases of the adnexa. Women who have never been pregnant are liable to have poorly developed round ligaments liable to tear away from the anchoring sutures. In these he opens the abdomen and shortens the round ligaments by one of the methods devised by Mann, Dudley or Wylie. He gives Mann's method the preference. Several of the patients have borne children both after the internal and external shortening of the round ligaments with good results. The writer now uses unabsorbable ligatures or sutures, always using plain catgut or chromicised catgut when a suture is used for a long period. He never has used any of the vaginal methods of fixation, not being pleased with them.

The uterus will now appear as far forward in either of the methods of shortening the round ligaments as it is in normal anteversion, but the pressure upon the posterior surface of the uterus will force the body into extreme anteversion and in a few months will appear perfectly normal in position.

### Third Day—Morning Session.

THE TECHNIQUE OF THE DRY METHOD

was the title of a paper by Dr. Edwin Walker, of Evansville, Ind., in which he said that by the dry method is meant a technique in which no water or other fluid is used. This does not apply to the preparation before the operation. After the first stroke of the knife until the wound is closed, not a drop of water is used. The writer has employed it exclusively for several years with good results. The technique is as follows: nurses are instructed to use every precaution to prevent soiling the hands in septic cases and to thoroughly disinfect the hands after any suspicion of contamination. Every instrument used is sterilised before it is put away. The hands are scrubbed thoroughly with a brush, with liquid soap (equal parts of green soap, glycerin and alcohol), and repeatedly rinsed in sterile water. They are then wiped off with alcohol, dipped for two minutes in bichlorid solution, 1 to 1000, and then washed off with salt solution. The field of operation is similarly prepared, except that the scrubbing is repeated daily for two or three days before the operation, and a soap poultice used at night. The instruments are boiled in soda solution for five to ten minutes, wrapped in towels or placed in metal boxes, which are opened only at the time of operation. Plain gauze is used for every purpose, except packing the uterus, and in rare instances where drainage is used iodoform gauze is preferred. The silk ligatures are wrapped on spools and placed in glass boxes, and the silkworm gut and silver wire in long glass tubes and all sterilised by steam, as are the dressings. The plain catgut is boiled in alcohol and the chromicised is prepared after Edebohl's method. After the patient is placed on the table the dressing, which consists usually of plain gauze, is removed from the field of operation, and sterilised towels adjusted as usual. The instruments are unwrapped, and everything is in readiness. The sponges are used dry and thrown away when soiled. The flat sponges in the abdomen are also used dry and are provided with a cord which is clamped with a pincette. The latter is left outside, so that the sponge cannot be forgotten and left in the abdomen. The author has not had extensive experience in puerperal sepsis or general septic peritonitis, but would be inclined to use it in both these conditions, for it has proven satisfactory in sepsis following abortions and localised collections of pus in the abdomen.

### SURGICAL SHOCK AND HEMORRHAGE, WITH REFERENCE TO PREVENTION AND TREATMENT.

This paper was read by Dr. Walter B. Chase, of Brooklyn, N. Y., in which the author presented the following summary :

1. The treatment of shock should be preventive and curative, and to a large degree the indications for the former define the lines of treatment in the latter.

2. The proper exhibition of preventive measures includes a careful study of the functional activity and organic status of all important organs, and such treatment by hygienic, dietetic and therapeutic measures as will elevate the standard of bodily and mental health to a degree in which the maximum power of resistance may be produced and maintained.

3. Special emphasis should be given to lithemic and uremic excretion, and to the condition of the circulatory and nervous systems.

4. Knowledge as to inherited power of resistance to, and recovery from, serious disease and accidents, are of the highest value in determining the course of procedure and in estimating the chances for recovery after capital operations.

5. A supply of facilities and drugs for meeting all emergencies should be in constant readiness, with exact knowledge for the indications, dosage, physiologic and therapeutic effect of special heart tonics and stimulants, including strychnia, digitalis, nitroglycerin, and the like.

6. Limit the time of an operation to the shortest space compatible with thorough work and proper technique.

7. Save the patient from the shock of fear to the utmost, and in selected cases proceed to operation without informing the patient of your purpose.

8. In shock with hemorrhage supply the volume of venous and arterial loss by direct transfusion of normal salt solution into the patient's veins.

9. Bear in mind the influence position has on the circulation under both shock and hemorrhage, especially in anemic conditions of the cerebrospinal nerve-centers and the heart.

Dr. W. H. Wenning, of Cincinnati, Ohio, read a paper on

PLACENTA PREVIA, WITH SPECIAL REFERENCE TO TREATMENT.

(Published in full, see page 175.)

A paper entitled

COMPLETE HYSTERECTOMY AFTER INJURY DURING PARTURITION AND CESAREAN SECTION WITH REPORT OF CASES, was read by Dr. Joseph H. Branham, of Baltimore.

The first patient was 24 years of age, female, married and had been pregnant for the third time. As the pelvic contracture was so marked there was no hope of a living child being born naturally ; the child was delivered by Cesarean section. The patient lost considerable blood per vaginam after the operation and rapidly developed symptoms of septic infection. She died two days and a half after the operation. The question arose as to whether Cesarean section or symphysiotomy was the better procedure. The essayist thinks the latter would have given the best chances for recovery, but as infection had already taken place, in all probability the final result would have been the same and even craniotomy, under the circumstances, would likely have proved fatal.

The second case was one of rupture of the uterus. The mortality of this condition was said to be 80 per cent. The accident may occur at any time from the third month of gestation until the termination of pregnancy. The causes and prominent symptoms of rupture of the uterus were detailed. *Treatment:*—When symptoms of uterine rupture occur, delivery should be completed as rapidly as possible by the use of whatever means may best bring about the results. The child nearly always dies in a few minutes, so that only the mother should be considered. If the child has escaped into the abdominal cavity immediate laparatomy is indicated. After the child had been delivered two methods of procedure are recommended by authority. One is the closing of the tear by packing with iodoform wicking and drainage of the parts with gauze. The other, which in complete ruptures is often more popular, is to do a laparatomy, cleansing the peritoneal cavity. In suitable cases close the tear by the Sänger method. If the tears are very extensive and there is a strong probability of uterine infection, a Porro operation or complete hysterectomy is the best method. The preventive method consists in the early relief of difficult labor by suitable operative interference instituted before the uterus has become very thin and damaged by long continued pressure between the presenting part and the bones of the pelvis.

Dr. X. O. WERDER, of Pittsburg, read a paper on

TONIC AND SPASMODIC INTESTINAL CONTRACTIONS, WITH REPORT OF CASES, in which he reviewed five cases, one by Dr. Murphy, two by Dr. Long, and two of his own, reported by Dr. Long at the Richmond, Va., meeting of the association and adds some additional cases of a similar condition that he has observed subsequently. He referred to cases reported by L. Heidenhain and applied the term enterospasm, dividing the cases according to their nature and severity into spasmodic and tonic or tetanic forms. He considers that they are preversions of normal peristalsis, due to a reflex chemical irritation exerted at the seat of contraction (either on the mucous or serous surfaces of the bowel) or elsewhere in the alimentary canal or abdomen and shows that they assume surgical importance when, in the spasmodic varieties, they simulate neoplasms, as in three cases of that variety reported ; or when they cause obstruction to the fecal current, becoming true cases of dynamic ileus, as in the five cases above referred to. Regarding the accuracy of diagnosis in these cases there can be but little doubt. Dr. Long's cases had undoubted symptoms of intestinal obstruction that varied in intensity over quite a long period. Operation revealed firm contractions of bowel. Careful search failed to show any other cause for the symptoms. Dr. Murphy's case, previously treated for several attacks of lead colic, operated on for intestinal obstruction of several days' duration, nothing was found but firm contraction of bowel. It relaxed after exposure to air. Three hours later spontaneous bowel movements occurred. Dr. Werder's first case gave history of previous attacks following ingestion of articles of diet made of milk. Simple salpingo-oöphorectomy for simple sarcoma. Clean case. Excellent condition for first six days. Onset of attack sudden and followed ingestion of egg-nogg by an hour or so. Symptoms of partial intestinal obstruction that later became complete, plus depressing defects of some toxic agents, were noted. Especial attention was called to two rational but misleading symptoms—namely, the occasional expulsion of gas for first three days, (that is, till patient was in collapse two days before death,) and an abdomen that was soft, flat and not tense or tender. Violent peristalsis persisted till death. Autopsy four hours later showed peritoneum everywhere glistening, normal. Pedicle covered by normal endothelium. No adhesion; no exudate; nothing abnormal could be found, except firm contraction of lower 55cm. of ileum and whole large intestine, and sacculation of section of bowel next above it. Cause of contraction attributed, as a probability, to tyrotoxicon absorption; death of patient, to intensified absorption of tyrotoxicon and other toxins forcibly retained in bowel. His second case followed vaginal hysterectomy for small fibroid. Symptoms of obstruction began earlier and were more pronounced than in the preceding case. Peristalsis persisted till death at the end of the fourth day. Autopsy two hours later showed sigmoid and ileum adherent to vaginal vault for an extent of three inches. All peritoneum visible from above was normal in appearance. No exudate. Bowel extending from seat of adhesion of ileum to valve and from adhesion of sigmoid to anus, firmly contracted. Sacculated bowel above. Plastic lymph at seat of adhesions and at the parts exposed to vaginal gauze and clamps. Two cc. of fluid found there. No pus visible to naked eye. No microscopic examination. Cause of contraction, chemical and mechanical irritation applied to serous surface; cause of death, possibility of mild sepsis combined with absorption of toxin from intestinal canal.

Of the spasmodic variety, the first case showed a sausage-shaped mass, three inches long and one inch thick at pylorus, with limited mobility and mapped out at each examination by several careful observers—not present when under ether and abdomen cleansed. Abdomen opened, pylorus and duodenum delivered; looked and felt normal. On manipulation they contracted firmly and were as hard as a finger, but only to relax in two or three minutes. This was repeated several times in full view of all present. In second case, contraction found accidentally when doing a suspensio-uteri. In third and fourth cases consulted for presence of tumor. Found firm contractions of bowel near umbilicus that resembled a neoplasm. They relaxed and contracted again several times during an examination that lasted fifteen or twenty minutes, always reappearing at the same place. Condition recognised and operation advised against. These patients were all neurotic and complained of quivering and commotion at seat of trouble, as well as of dyspepsia and obstinate constipation.

### DYNAMIC ILEUS FOLLOWING OPERATIONS INVOLVING THE ABDOMINAL CAVITY, WITH REMARKS ON ADYNAMIC ILEUS.

By Dr. Frederick Blume, of Allegheny, Pa. (Published in full, see page 161.)

### Third Day—Afternoon Session.

Dr. Albert Goldspohn, of Chicago, contributed a paper on

### THE FATE OF OVARIES IN CONNECTION WITH RETROVERSION AND RETROFLEXION OF THE UTERUS.

He drew the following conclusions: (1) in all cases of retroversion and retroflexion of the uterus a knowledge of the ovaries as to their location, mobility and general physical condition should comprise an essential part in the diagnosis, as determining largely the nature and urgency of the treatment; (2) the welfare of ovaries, in general, demands such a degree of anterior inclination of the longitudinal axis of the uterus as will enable intraäbdominal pressure to bear upon the posterior surface of the organ and thereby to act in unison with its other supports to retain it and its adnexa in normal position and function; (3) inasmuch as in the female pelvis, as well as elsewhere in the human body, the natural and considerable abilities of healthy tissues to defend themselves against microbic invasion (infection) are lowered or annuled in direct proportion to any degree of mechanical embarrassment of the venous circulation in the tissues or organs, it behooves gynecologists especially to be alert in recognising and correcting all material anomalies in place or posture of the female generative organs or in securing to them their normal freedom.

Dr. Walter B. Dorsett, of St. Louis, Mo., read a paper entitled

### THE ADMINISTRATION OF PHOSPHATE OF STRYCHNIA DURING GESTATION.

The following observations have been made by him in the use of this drug during the gestation of weak and debilitated patients:

A good appetite and good assimilation are obtained in the general weakness and debility of the anemic. Constipation is relieved, the patient is built up and placed in a good condition to pass through the ordeal of labor. The uterus contracts promptly after the third stage of labor, and the use of ergot is entirely dispensed with. If he finds it necessary to use the forceps, the patient is given a hypodermic injection of 1-30 grain sulphate or phosphate of strychnia as soon as the anesthetic is commenced, but no ergot is ever used. After the continuous use of the phosphate of strychnia, the uterus contracts promptly after the second stage of labor and in many cases the application of Credé's method of expression of the placenta is not needed to bring it away and no postpartum hemorrhages have occurred. The frequently observed chilliness or rigors, which in the majority of cases follow labor, has been noticed in but few cases. He has used strychnia for some time in his abdominal surgery, for the purpose of preventing shock and to control the pulse in the operations, and in this way was led to its use in obstetrics. As phosphorus and strychnia are remedies used in the treatment of rachitis with good results, would it not be the remedy during the gestation of the rachitic fetus ?

The remainder of this session was devoted to the exhibition of pathologic specimens, with histories and photographs of the same. Specimens were presented and the histories of cases related by Drs. Vander Veer, Dorsett, Macdonald, Ross, McMurtry, Chase, Smith, Hughes and others.

### Fourth Day—Morning Session.

Dr. Geo. S. Peck, of Youngstown, Ohio, read a paper in which he reported

### FIFTY-TWO CASES ILLUSTRATING HIS PERSONAL EXPERIENCE WITH THE MEDICAL AND SURGICAL TREATMENT OF APPENDICITIS.

He said there were four cardinal symptoms which will almost invariably insure a correct diagnosis, if they occur in the order given : (1) sudden severe pain in the abdomen, generally of a colicky nature, located in any part or extending over the entire abdomen ; (2) always nausea and frequently vomiting ; (3) increased temperature ; (4) localised tenderness in the right iliac region. Some patients will have diarrhea, while others may be constipated. He had never failed to make a correct diagnosis when the four cardinal symptoms were present.

*Treatment.*—Surgeons differ in their methods of operating. Some advise in the acute suppurative form simple incision and evacuation of pus. If the appendix cannot be easily found it should be left drained and packed and then, in the interval between attacks, the appendix should be removed, always provided one can get the consent of the patient, which the speaker has found to be difficult. A few surgeons advise the liberation of all adhesions and the removal of the appendix in all cases of acute suppurative appendicitis and have reported good results. He believes it is the duty of every surgeon to make a complete operation in the vast majority of cases and thinks that the time is not far distant when surgeons will advise the breaking up of all adhesions, the removal of every diseased appendix, the closing of the incision as is now done in the operation for pyosalpinx, as advised by Morris, Price and McMurtry. In his last three cases he has followed this method and the results have been far beyond his expectations. Two of the cases were discharged in three and one in four weeks.

Dr. Lewis S. McMurtry, of Louisville, Ky., followed with a paper entitled

THE OPERATION ITSELF IN APPENDICITIS.

He considered the subject under the following headings : (1) the incision ; (2) dealing with adhesions and with abscesses ; (3) removal of the appendix ; (4) drainage and isolation of the peritoneum by gauze.

Concerning the incision three important considerations must be observed : (1) to obtain easy access to the caput coli with sufficient working space ; (2) to secure all natural advantages to facilitate drainage ; (3) to do the least damage possible to the parietal structures incised, in order that firm union may be secured and hernia thereby prevented. The early operations for appendicitis were mostly in extreme cases wherein suppuration had obtained and in cutting down into an abscess and evacuation and draining the same. For this purpose the vertical incision was adopted and is yet practised by many surgeons. This incision does not, however, give as easy access to the appendix and to the outer and posterior areas adjacent thereto, which are so frequently involved, as does the oblique incision. A method of dividing the abdominal wall by a combination of incision and blunt dissection has been described by McBurney and commended by many writers on the surgery of the appendix. The incision was described at length.

*Dealing with adhesions and abscesses.*—In dealing with adhesions and abscesses the same general rules of surgical treatment should be observed in appendicitis as in similar conditions affecting other organs enclosed within the peritoneum. Whenever practicable, adhesions should be separated, abscesses emptied, disintegrated structures composing foci of infection removed and cleansing and drainage secured by measures of assured efficiency.

*Removal of the appendix.*—In his early operations he ligated the appendix with the meso-appendix, using fine sterilised silk, cut away the appendix and applied pure carbolic acid to the stump. Later he adopted the modern method of transfixing the meso-appendix at its base, cutting it away, stripping back a cuff of peritoneum from the appendix down to its junction with the cecum, ligating the appendix with fine silk and cutting it away, sterilising the stump, invaginating the stump into the cecum and covering with peritoneum by careful stitching after the Lembert method. Nothing in the progress of healing or in ultimate results indicated any advantage of the latter method over the former simple ligature, excision and cauterisation.

Finally, Dr. McMurtry discussed drainage and isolation of the peritoneum by gauze.

Dr. THOMAS J. MAXWELL, of Keokuk, Iowa, contributed a paper on Senile Irritable Uterus. In three cases he was unable to relieve this condition by tentative treatment and finally resorted to hysterectomy with complete success in all of them.

The election of officers resulted as follows :

President, Dr. Charles A. L. Reed, of Cincinnati, Ohio ; vice-presidents, Dr. Richard Douglas, Nashville, Tenn., and Dr. Walter B. Dorsett, St. Louis, Mo. ; secretary, Dr. Wm. Warren Potter, of Buffalo, N. Y.; treasurer, Dr. X. O. Werder, of Pittsburg, Pa. Place of meeting, Pittsburg, Pa., September 20, 21 and 22, 1898.

After introducing and adopting resolutions of thanks for courtesies extended, the association adjourned.

---

TYPHOID FEVER.—Keep the patient in bed until, for an entire week, the temperature has been normal. Keep him on a sterilised liquid diet as long as he remains in bed. At the beginning of the disease give ten grains of calomel on alternate days and also give one grain of carbolic acid and three drops of tincture of iodine every four hours during the entire illness.—*Medical Record.*

